# Cost‐effective production of tag‐less recombinant protein in *Nicotiana benthamiana*

**DOI:** 10.1111/pbi.13040

**Published:** 2018-12-08

**Authors:** Md Reyazul Islam, Ju‐Won Kwak, Jeon‐soo Lee, Sung‐Wook Hong, Md Rezaul Islam Khan, Yongjik Lee, Yoontae Lee, Seung‐Woo Lee, Inhwan Hwang

**Affiliations:** ^1^ Division of Integrative Biosciences and Biotechnology Pohang University of Science and Technology Pohang Korea; ^2^ Department of Life Science Pohang University of Science and Technology Pohang Korea

**Keywords:** plant‐based expression system, *Nicotiana benthamiana*, human interleukin‐6, cellulose‐binding domain, proteolytic tag removal, bdSUMO, bdSENP1

## Abstract

Plants have recently received a great deal of attention as a means of producing recombinant proteins. Despite this, a limited number of recombinant proteins are currently on the market and, if plants are to be more widely used, a cost‐effective and efficient purification method is urgently needed. Although affinity tags are convenient tools for protein purification, the presence of a tag on the recombinant protein is undesirable for many applications. A cost‐effective method of purification using an affinity tag and the removal of the tag after purification has been developed. The family 3 cellulose‐binding domain (CBM3), which binds to microcrystalline cellulose, served as the affinity tag and the small ubiquitin‐related modifier (SUMO) and SUMO‐specific protease were used to remove it. This method, together with size‐exclusion chromatography, enabled purification of human interleukin‐6 (hIL6) with a yield of 18.49 mg/kg fresh weight from leaf extracts of *Nicotiana benthamiana* following *Agrobacterium*‐mediated transient expression. Plant‐produced hIL6 (P‐hIL6) contained less than 0.2 EU/μg (0.02 ng/mL) endotoxin. P‐hIL6 activated the Janus kinase‐signal transducer and activator of transcriptional pathways in human LNCaP cells, and induced expression of *IL‐21* in activated mouse CD4^+^ T cells. This approach is thus a powerful method for producing recombinant proteins in plants.

## Introduction

The commercialization of recombinant proteins is now a mainstream area in biotechnology. Currently, many different systems, including bacteria, yeasts and animal cells, are used to produce recombinant proteins (Berlec and Štrukelj, 2013; Gerngross, 2004). Plants have recently received considerable attention as a platform for protein production due to their unique characteristics, which include high scalability and low capital investment required for infrastructure. Moreover, protein production in plants is compatible with green technology, pathogen and endotoxin‐free, and, most importantly, cost‐effective and widely acceptable to society (Holtz *et al*., 2015; Islam *et al*., 2018; Schillberg *et al*., 2003).

Several technologies have been developed to enable protein production in plants. The most important of these is the design of vectors allowing high levels of gene expression in plants (Mortimer *et al*., 2015; Regnard *et al*., 2010; Werner *et al*., 2011). There are many more possible options for gene expression in plants than in animal cells and bacteria. The most powerful is transient expression following *Agrobacterium*‐mediated gene transfer into host plants *via* RNA virus‐mediated RNA amplification or DNA virus‐based gene amplification. Another important approach is chloroplast transformation, which integrates the target gene into the chloroplast genome and thereby leads to extremely high levels of protein production (Staub *et al*., 2000; Verma and Daniell, 2007; Zhang *et al*., 2017); the transgenic approach, which integrates foreign genes into the plant nuclear genome, is also widely used (Lee *et al*., 2015; Sohn *et al*., 2018; Twyman *et al*., 2002). Many different strategies using transgenic plants can be employed as recombinant proteins can be obtained from either whole plants or cell cultures (Holtz *et al*., 2015; Rosales‐Mendoza and Nieto‐Gómez, 2018; Tekoah *et al*., 2015). Carrot cell cultures have been used to produce taliglucerase‐α which is used to treat Gausher's disease. If whole plants are used, different tissues can be employed to produce recombinant proteins.

Protein purification is another critical step in protein production. This step largely determines the cost of recombinant protein‐based products and there is thus an urgent need to develop better and more convenient purification methods. Affinity‐based purification is one of the most convenient current methods. Antibodies, for example have a highly specific binding affinity to protein A or protein G produced in bacteria (Björck and Kronvall, 1984; Huse *et al*., 2002); high mannose‐containing proteins have a high binding affinity to ConA produced in plants (Bereli *et al*., 2005); and certain viral proteins or virus‐like particles have specific affinities to heparin sulphate (Gieseler *et al*., 2017). In most cases, however, cellular proteins lack specific affinities to other molecules that can be exploited during their purification. In such cases, a specific binding domain can be fused to the target protein as an affinity tag. GST and maltose binding protein are tags with specific affinities to glutathione and amylose respectively (Arnau *et al*., 2006; Pina *et al*., 2014), and an artificial affinity tag, His×6, has a high affinity to Ni^2+^‐NTA (Cheung *et al*., 2012; Pina *et al*., 2014). Most affinity tags are expensive, however, which increases the cost of protein production (Fong *et al*., 2010). Current methods of purifying proteins produced by plants largely rely on these traditional and expensive methods, thereby nullifying the advantage of using plants to reduce the cost of protein production. Moreover, plants have a highly complex protein pool, which makes the purification of proteins from plant extracts more challenging. There is thus a high demand for a new and cost‐effective method of protein purification from plants.

Recently, cellulose‐binding domains (CBDs), which have a high binding affinity to cellulose, an inexpensive biomaterial, have been used as affinity tags (Bayer *et al*., 1998; Sohn *et al*., 2018; Wan *et al*., 2011; You and Zhang, 2013). The CBDs can be divided into multiple groups and show specific binding to either the crystalline or amorphous regions of cellulose fibres.

Another challenge in the production of recombinant protein is the removal of the affinity tag after purification. Proteases such as thrombin, enterokinase (EK) or tobacco etch virus (TEV) protease, which recognize protease‐specific short peptide sequences, have been widely used for tag removal; however, most proteases, other than EK, leave extra residues on the target protein after cleavage, which is often undesirable (Arnau *et al*., 2006). The small ubiquitin‐related modifier (SUMO) protease was introduced to remove fused domains from the target proteins (Butt *et al*., 2005; Malakhov *et al*., 2004). SUMO proteases specifically recognize the tertiary sequence of the entire SUMO domain and cleave off a double‐glycine (GG) motif at its C‐terminus of the recognition site. This process leaves no extra residues on the target proteins. Moreover, as SUMO proteases recognize the SUMO domain, they are more highly specific than other proteases.

We have developed a method for purifying recombinant protein from plant extracts and subsequently removing the fusion domain from the target proteins using a SUMO domain and SUMO‐specific protease 1 (bdSENP1) from *Brachypodium distachyon*. The family 3 cellulose‐binding domain (CBM3), a CBD from *Clostridium thermocellum*, showed a high affinity to microcrystalline cellulose (MCC) beads and thus could be used as a specific purification tag for recombinant proteins expressed in plant cells. bdSENP1 specifically removed this affinity tag and the SUMO domain from target proteins bound to the surface of cellulose beads.

## Results

### Design of a recombinant gene for high level expression in plants

We considered two factors in developing a plant‐based recombinant protein production system: cost‐effective purification from plant extracts and removal of the tag from the target proteins. To ensure cost‐effective, high‐efficiency purification, we employed CBM3, a CBD, as an affinity tag. CBM3 binds to the surface of MCC (Hong *et al*., 2007) and thus inexpensive cellulose beads can be used as an affinity resin. CBDs have been previously used as affinity tags for purifying proteins from *Escherichia coli* and fungal extracts (Wan *et al*., 2011; Wang and Hong, 2014; You and Zhang, 2013).

To remove the affinity tag from the target proteins, we used bdSUMO and bdSENP1 from *Brachypodium distachyon* as a SUMO domain and SUMO‐specific protease, respectively (Frey and Görlich, 2014). The SUMO domain is often expressed as an N‐terminal domain of a polypeptide and can be released from the polypeptide *via* specific proteolytic cleavage by SUMO‐specific protease (Butt *et al*., 2005). The SUMO‐specific proteases remove all the residues at the cleavage site, together with SUMO domain (Malakhov *et al*., 2004), releasing the C‐terminal domain of the polypeptide with no extra residues.

**Figure 1:**
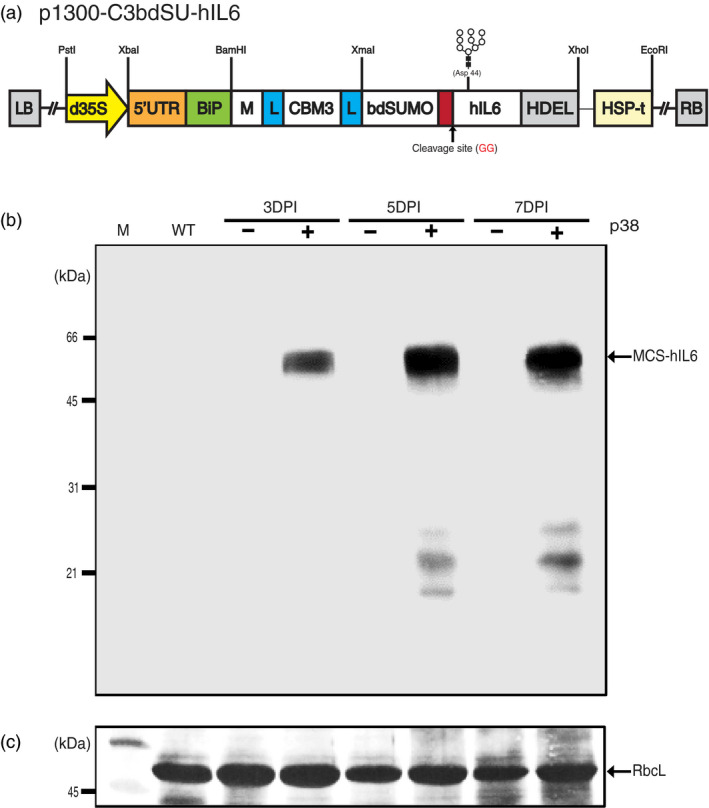
*Agrobacterium*‐mediated transient expression enables high levels of MCS‐hIL6 production in *Nicotiana benthamiana*. (a) Design of a chimeric hybrid construct for high level protein expression, purification, and affinity tag removal. The plant expression binary vector p1300‐C3bdSU‐hIL6 contains a chimeric hybrid construct consisting of various domains, including a plant codon‐optimized M domain, a cellulose‐binding domain (CBM3), a small ubiquitin‐related modifier (bdSUMO) derived from *Brachypodium distachyon*, and human interleukin‐6 (hIL6) flexible linkers (L). In addition, the BiP leader sequence and an ER retention signal HDEL were fused to the 5’ and 3’ ends of the chimeric construct, respectively. The chimeric construct was placed under the double enhancer‐containing CaMV 35S promoter (d35S) and a strong translational enhancer sequence (5′ UTR) followed by the HSP terminator (HSP‐t) from *Arabidopsis thaliana*. The N‐linked glycosylation site (Asp‐44) of hIL6 is indicated in gray. (b) Western blot analysis of MCS‐hIL6. *Nicotiana benthamiana* leaf tissues were harvested at the indicated time points after infiltration of p1300‐C3bdSU‐hIL6 with (+) or without (−) the p38 silencing suppressor. Total leaf extracts (30 μg) were separated using 12.5% SDS‐PAGE and analyzed by western blotting with anti‐IL6 antibody. (c) Coomassie brilliant blue (CBB) staining. A membrane identical to that used for western blot analysis was stained with CBB. The large subunit of the rubisco complex (RbcL) was used as a loading control. M: molecular weight standards; WT: wild‐type *N. benthamiana* leaf tissue extracts. The arrow indicates the position of MCS‐hIL6 fusion protein bands (60–65 kDa).

A previous study found that the endoplasmic reticulum (ER) accumulates high levels of recombinant proteins (Gomord *et al*., 1997; Kang *et al*., 2018; Sohn *et al*., 2018; Wandelt *et al*., 1992). Thus, to achieve a high level of expression, we used the BiP leader sequence to target the recombinant protein to the ER. In addition, ER localization can protect recombinant proteins from unwanted proteolytic cleavage by endogenous SUMO‐specific proteases present in the plant cytosol (Nayak and Müller, 2014). To enable protein accumulation in the ER, we added the ER retention signal, HDEL, to the C‐terminus (Pagny *et al*., 1999). We also included another important component, the highly mannosylated N‐glycan‐containing polypeptide (M) domain. The M domain, derived from human protein tyrosine phosphatase, receptor type C (CD45), has multiple N‐glycosylation sites and, when fused to a target protein, increases translation levels up to sevenfold (Kang *et al*., 2018). The M domain was inserted next to the BiP leader sequence, so it could be removed from the target protein, together with CBM3 and bdSUMO, by cleavage with bdSENP1 protease (Figure 1a).

Human interleukin‐6 (hIL6) was used as a target protein, as its activity could be easily tested *via* the Janus kinase‐signal transducer and activator of transcription (JAK‐STAT) pathway in animal cells (Yu *et al*., 2014). The different domains, BiP, M, CBM3, bdSUMO, hIL6 and HDEL, were fused to produce *BiP‐M‐CBM3‐bdSUMO‐hIL6‐HDEL* (*MCS‐hIL6*). A flexible linker (L), consisting of glycine‐glycine‐glycine‐glycine‐serine × 2, was inserted on either side of the CBM3 domain (Figure 1a).

### Optimization of MCS‐hIL6 expression in *Nicotiana benthamiana* leaves

We used transient expression induced by *Agrobacterium*‐mediated infiltration to express recombinant proteins in *Nicotiana benthamiana*. To determine the optimal conditions for high level expression of *MCS‐hIL6* in *N. benthamiana* leaves, plant leaf tissues were infiltrated with *Agrobacterium* culture harbouring *MCS‐hIL6* singly or together with an *Agrobacterium* culture harbouring p38 of the *Turnip crinkle virus* silencing suppressor (Qu *et al*., 2003). Leaves were harvested 3, 5 or 7 days post‐infiltration (DPI), and protein extracts from leaf tissue were analysed using Western blotting with anti‐IL6 antibody (Figure 1b). No specific band was detected by anti‐IL6 antibody in leaf tissue infiltrated with MCS‐hIL6 alone; by contrast, anti‐IL6 antibody‐positive bands were detected at a molecular weight range between 60 and 65 kDa in leaf tissue infiltrated with MCS‐hIL6 together with p38. The intensity of the bands increased between 3 and 5 DPI, and then plateaued at 7 DPI. In addition, a few bands of less than 31 kDa were detected at 5 and 7 DPI, indicating that a small portion of MCS‐IL6 had degraded.

### MCS‐hIL6 binds strongly to MCC beads

**Figure 2:**
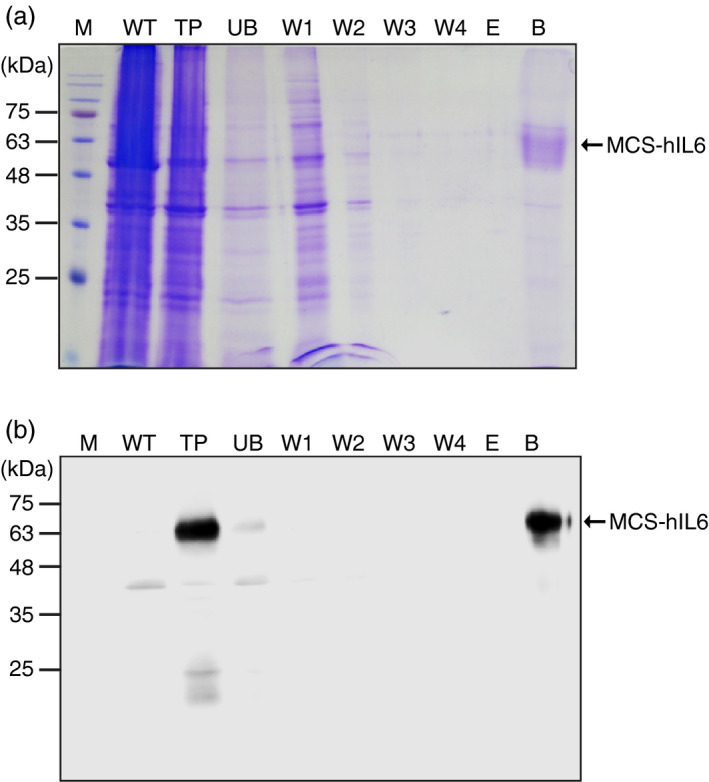
CBM3 in MCS‐hIL6 irreversibly binds to microcrystalline cellulose (MCC) beads. (a, b) Coomassie brilliant blue (CBB) staining and western blot analysis. Total plant leaf extracts (TP) were incubated with MCC beads at 4 °C for 1 h. Proteins in the supernatants were collected as the unbound (UB) fraction. MCC beads were washed four times and each wash‐off fraction (W1 to W4) was collected separately. After the fourth wash, the MCC beads were treated with 80% glycerol and the supernatant was collected (E fraction). Finally, the MCC beads were boiled in SDS loading buffer and the supernatant was collected (B fraction). These fractions were analyzed using 12.5% SDS‐PAGE followed by CBB staining (a) or western blot analysis with anti‐IL6 antibody (b). M: pre‐stained molecular weight standard; WT: wild‐type *N. benthamiana* leaf tissue extracts. Arrows indicate the position of MCS‐hIL6 (60–65 kDa).

We examined whether MCS‐hIL6 could be purified using MCC beads. We first determined the binding ability of CBM3 to MCC using CBM3‐tagged MerP (CBM3:MerP), a recombinant protein expressed and purified from *E. coli*. We found that 20 μg CBM3 could bind to 10 mg MCC beads (2 mg/g or 2 mg/3 mL bead‐volume; Figure S1). Based on this result, and the level of expression of MCS‐hIL6 in *N. benthamiana*, total protein extracts were incubated with MCC beads at a 10 : 1 (v/v) ratio. Most plant proteins were detected in the unbound (UB) and first wash‐off (W1) fractions. No plant proteins were detected in the W3 to W4 fractions, indicating that weakly bound proteins were removed by two washing steps. We attempted to elute proteins from the MCC beads. A previous study showed that proteins bound to MCC by CBD could be eluted using 80% glycerol, 40%–100% ethylene glycol and 0.1 m glycine (Wan *et al*., 2011). We treated the MCC beads with 80% glycerol to determine whether MCS‐hIL6 protein was released; no proteins were detected in the eluent, indicating that CBM3 was tightly bound to MCC. MCS‐hIL6 was released from MCC, however, by boiling in SDS‐reducing buffer (Figure 2a,b). The Coomassie brilliant blue (CBB)‐stained SDS gel (Figure 2a) suggested high purity in the MCS‐hIL6 released from MCC beads by boiling, indicating that this approach produced rather pure protein.

### bdSENP1 cleaves MCC‐immobilized bdSUMO domain and releases C‐terminally fused hIL6

We examined whether proteolytic digestion with His:bdSENP1 *in vitro* could release hIL6, fused at the C‐terminus region, from the chimeric protein, MCS‐hIL6, immobilized on MCC beads. Previous studies showed that bdSENP1 is highly active at a wide range of temperatures *in vitro* (Frey and Görlich, 2014). His:bdSENP1 was expressed in *E. coli* and purified by Ni^2+^‐NTA affinity chromatography using the N‐terminal His‐tag (Figure S2).

**Figure 3:**
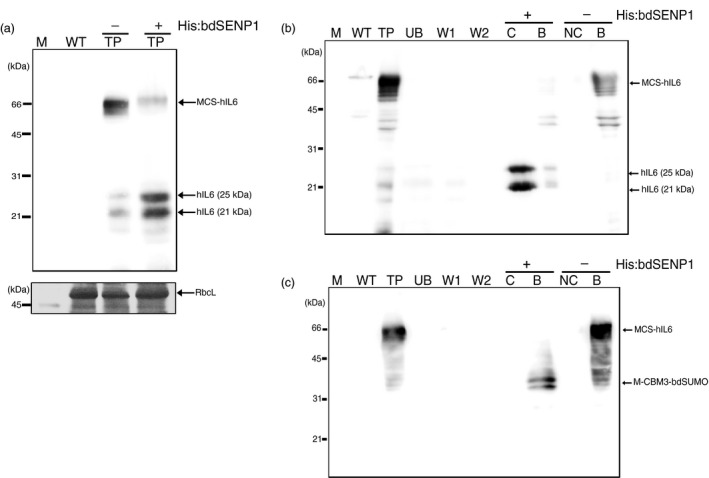
MCS‐hIL6 is cleaved by His:bdSENP1 both in crude leaf extracts and when immobilized on microcrystalline cellulose (MCC). (a) bdSENP1‐mediated cleavage of bdSUMO in MCS‐hIL6. Total leaf extracts were treated with (+) or without (−) His:bdSENP1 and analyzed by western blotting with anti‐IL6 antibody. The large subunit of the rubisco complex (RbcL) stained with CBB was used as a loading control. (b, c) bdSENP1‐mediated cleavage of bdSUMO in MCS‐hIL6 immobilized on MCC beads. Total protein extracts were incubated with MCC beads. After binding, the MCC beads were washed twice and treated with (+) or without (−) His:bdSENP1. Proteins in the supernatant and MCC bead fractions were collected separately and analyzed by western blotting with anti‐IL6 antibody (b) or anti‐CBM3 antibody (c). M: molecular weight standards; WT: wild‐type total leaf extracts; TP: total leaf extracts; UB: unbound fraction; W1 and W2: first and second wash‐off fractions, respectively; S: supernatant after His:bdSENP1 treatment; NS: supernatant without His:bdSENP1 treatment; B: proteins released from MCC beads by boiling.

We determined whether His:bdSENP1 could digest MCS‐hIL6 by recognizing bdSUMO domain in the recombinant protein *in vitro*. Total protein extracts were incubated with His:bdSENP1 *in vitro* and the protein samples were analysed using Western blotting with anti‐IL6 antibody (Figure 3a). MCS‐hIL6‐specific bands were detected at 60–65 kDa without treatment with His:bdSENP1; by contrast, following His:bdSENP1 treatment, hIL6‐specific bands were detected as a doublet at 21 and 25 kDa resulting from a difference in the degree of N‐glycosylation (see below in Figure 5), indicating that MCS‐hIL6 had been digested by His:bdSENP1.

Next, we examined whether MCC‐immobilized MCS‐hIL6 was digested by His:bdSENP1 *in vitro*. Total protein extracts from *N. benthamiana* leaf tissue at 5 DPI were incubated with MCC beads. The beads were washed with washing buffer four times, and then His:bdSENP1 in reaction buffer was added and the beads were incubated at 4 °C for 6 h. Proteins were recovered from the supernatant. The MCC beads were collected separately; proteins remained bound to the beads were released by boiling in SDS‐reducing buffer. The proteins were analysed by Western blotting with anti‐IL6 and anti‐CBM3 antibodies (Figure 3b,c). A 60–65 kDa MCS‐hIL6‐specific band was detected in the total protein extracts; in contrast, after incubation with His:bdSENP1, hIL6‐specific bands were detected at 21 and 25 kDa, indicating that His:bdSENP1 cleaved MCS‐hIL6 bound to the MCC beads to release hIL6. The anti‐CBM3 antibody detected a new 39 kDa protein species in the MCC bead fraction (Figure 3c), indicating that a 39 kDa fragment remained bound to the beads. This 39 kDa protein species was the predicted size of the N‐terminal region containing the three domains, M, CBM3 and bdSUMO domain. These results suggested that bdSENP1 could digest the immobilized form of bdSUMO domain‐containing recombinant proteins on MCC beads.

### Recombinant hIL6 without an affinity tag can be obtained at high purity with low levels of endotoxin contamination

**Figure 4:**
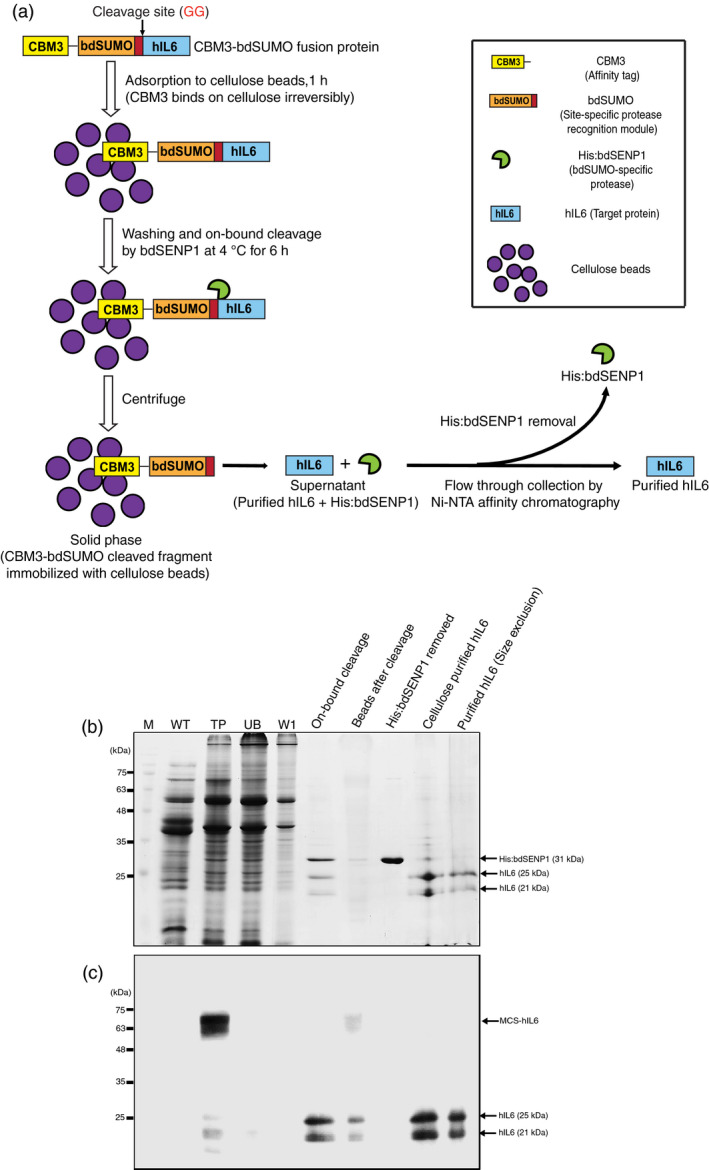
Production of hIL6 from *N. benthamiana* leaf tissue *via* MCC‐based affinity purification, SUMO‐based removal of the affinity tag and size‐exclusion chromatography. (a) Schematic illustrating purification of CBM3‐bdSUMO‐tagged protein and removal of the affinity tag. (b, c) Production of hIL6. Fresh leaf tissues (40 g) were homogenized in extraction buffer and total protein extracts were prepared. The total protein extracts were incubated with MCC beads at a 10: 1 ratio. After incubation at 4 °C, the supernatant was collected (unbound fraction, UB) and the MCC beads were washed four times in washing buffer (W). The MCC beads were treated with 10 μg His:bdSENP1 at 4 °C for 6 h. The supernatant was collected (On‐bound cleavage) and His:bdSENP1 was removed by passing the supernatant through a Ni^2+^‐NTA affinity column (His:bdSENP1 removed). The flow‐through fraction containing hIL6 was collected (cellulose purified fraction) and applied to the size‐exclusion column. Fractions were collected from the size‐exclusion column. The fractions obtained at each step were analyzed using SDS‐PAGE followed by CBB staining (b) or western blot analysis with anti‐IL6 antibody (c). M: pre‐stained molecular weight standards; WT: wild‐type total leaf extracts; T: total leaf extracts of MCS‐hIL6; UB: unbound fraction; W: wash‐off solution.

On the basis of the results shown in Figures 2 and 3, we established a protocol for obtaining tag‐free target protein from plant extracts (Figure 4a). To test this protocol, we prepared extracts from 40 g *N. benthamiana* leaf tissue harvested at 5 DPI. Total soluble protein extracts containing MCS‐hIL6 were incubated with MCC beads. The MCC beads with bound proteins were washed four times and incubated with purified His:bdSENP1 protease at 4 °C for 6 h. After digestion, the supernatant was passed through a Ni^2+^‐NTA agarose column to remove His:bdSENP1 by Ni^2+^‐NTA affinity column chromatography. His:bdSENP1 was removed from the supernatant, yielding rather pure hIL6 (Figure 4b). The CBB‐stained gel showed 31 kDa of His:bdSENP1.

To further improve the purity of hIL6, we employed size‐exclusion chromatography using a HiLoad 16/600 Superdex 200 pg column. The flow‐through fractions obtained from the Ni^2+^‐NTA affinity column were applied to the HiLoad 16/600 Superdex 200 pg column and fractions were collected. These fractions were analysed by Western blotting using anti‐IL6 antibody. Two protein bands at 21 and 25 kDa were detected. The size‐exclusion chromatography elution profiles and Western blot analysis indicated that hIL6 corresponded with the major first peak (elution time: 71.91 min) and the second peak (elution time: 74.11 min) at 280 nm absorbance (Figure S3A,B).

To further confirm hIL6 purity, proteins in the fractions were concentrated, separated by SDS‐PAGE and stained with CBB (Figure 4b). The same proteins were also analysed by Western blotting with anti‐IL6 antibody (Figure 4c). The CBB‐stained gel showed two major bands at 21 and 25 kDa, but no other visible bands.

The quantitative analysis of purified hIL6 was performed using the Bradford protein assay with bovine serum albumin (BSA) as a standard. We also estimated the amount of hIL6 (0.5–3 μg) by comparing the intensity of CBB staining of protein bands with the intensity obtained from different BSA (0.5–4 μg) standards following separation on 12.5% SDS‐PAGE (Figure S4A). To confirm the quantification, we compared the intensity of protein bands of hIL6 purified from plants with that of commercial hIL6 produced from *E. coli* using Western blotting with anti‐IL6 antibody (Figure S4B,C). The yields of hIL6 were estimated to be approximately 18.5 mg/kg fresh weight (FW) of *N. benthamiana* leaves at about >95% purity, according to the SDS‐PAGE analysis.

One major advantage of using plants to produce recombinant proteins is the potential for low levels of endotoxin such as (lipopolysaccharide; LPS) compared to production in *E. coli*. As we used *Agrobacterium*‐mediated transient expression to express hIL6 in plants, we examined the endotoxin content in hIL6 purified from plant tissue using the chromogenic kinetic method based on the *Limulus* amebocyte lysate assay (LAL‐test). hIL6 purified from plants contained <0.20 EU/μg (<0.02 ng/mL) endotoxin (Figure S5A,B), indicating that very low levels of LPS were present in the purified proteins despite the use of *Agrobacterium* to express hIL6.

### N‐glycosylated hIL6 produced in plants is biologically active

**Figure 5:**
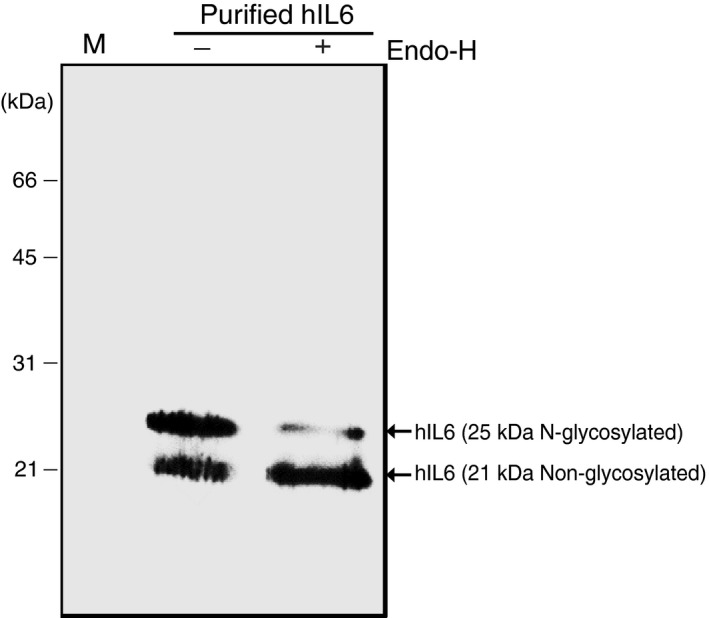
Plant‐produced hIL6 has a high mannose‐type N‐glycosylation. Purified hIL6 was treated with (+) or without (−) endo‐H and analyzed by western blotting with anti‐IL6 antibody.

We examined the nature of the hIL6 doublet with molecular masses of 21 and 25 kDa to determine whether hIL6 was modified by N‐glycosylation. hIL6 has one potential N‐glycosylation site at Asp44 (Figure 1a). MCS‐hIL6 has an ER retention signal at the C‐terminus and thus if hIL6 contains an N‐glycan moiety, it should be the high mannose type and sensitive to endoglycosidase H (endo‐H). Purified hIL6 was treated with endo‐H and analysed by Western blotting with anti‐IL6 antibody (Figure 5). Endo‐H treatment significantly reduced the amount of 25 kDa hIL6 and concomitantly increased the amount of 21 kDa hIL6, indicating that 25 kDa hIL6 was N‐glycosylated. This suggested that the two hIL6 bands represented the N‐glycosylated and non‐glycosylated forms.

**Figure 6:**
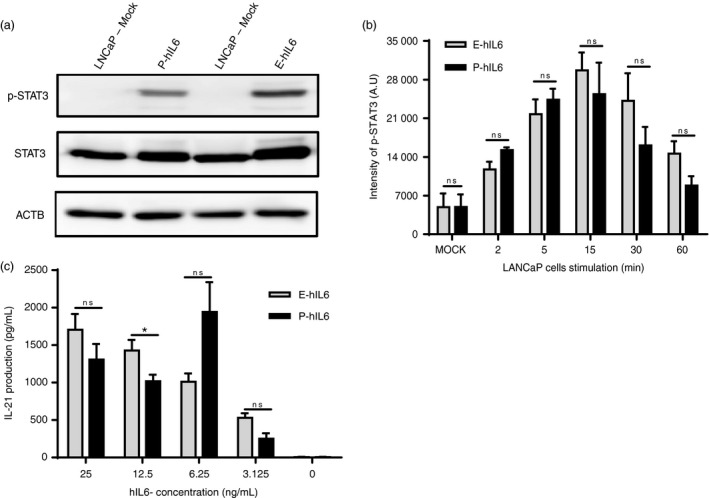
Plant‐produced hIL6 is active in the STAT3 signalling pathway in animal cells. (a, b) Phosphorylation of STAT‐3 (p‐STAT3) and quantification of p‐STAT3 levels. Total protein extracts from LNCaP cells, treated with plant‐produced hIL6 (P‐hIL6) or commercial *E. coli*‐produced hIL6 (E‐hIL6) for 30 min (a) or for the indicated period of time (b), were analyzed by western blotting with anti‐p‐STAT3 (phosphorylated STAT3), anti‐STAT3, or anti‐β‐actin (ACTB) antibodies. β‐actin was used as a loading control. The intensity of p‐STAT3 bands was measured using ImageJ software to quantify the amount of p‐STAT3. Three independent experiments were performed. Error bars, SEM (*n* = 3). Statistical analysis was performed using the Student's t‐test between P‐hIL6 and E‐hIL6 at the indicated time points; ns: no significant difference. (c) IL6‐induced IL‐21 expression. FACS‐sorted CD25^−^ total CD4^+^ T cells from mouse splenocytes were cultured in the presence of different quantities of P‐hIL6 or E‐hIL6. IL‐21 production was measured by ELISA after 3 days of treatment. The data are the means ± SEM (*n* = 3). Data were analyzed using the Student's *t*‐test; *: *P* < 0.04; ns: no significant difference.

We next examined whether plant‐produced hIL6 (P‐hIL6) was biologically active. As hIL6 activates the Janus kinase‐signal transducer and activator of transcription (JAK‐STAT) pathway (Chen *et al*., 2000; Yu *et al*., 2014), we examined the degree of phosphorylation of the signal transducer and activator of transcription 3 (STAT3) in an androgen‐sensitive human prostate adenocarcinoma (LNCaP) cell line (Chen *et al*., 2000). The first step in the pathway is the binding of IL6 to the IL6 receptor, which interacts and induces the dimerization of signalling receptor subunit glycoprotein 130 (gp130). This subsequently triggers an intracellular signalling cascade through the activation of gp130‐associated protein tyrosine kinases of the JAK families and activation of STAT3 by phosphorylation (Yu *et al*., 2014). We treated LNCaP cells with 150 ng/mL P‐hIL6 and observed phosphorylation of STAT3 (p‐STAT3) (Figure 6a). As a positive control, we used hIL6 produced in *E. coli* (E‐hIL6). The level of STAT3 phosphorylation was slightly lower in P‐hIL6 than in E‐hIL6. We then performed a time‐course study of STAT3 phosphorylation to compare the degree of activation. Following treatment with either P‐hIL6 or E‐hIL6, the level of STAT3 phosphorylation increased with time up to 15 min and then decreased (Figure 6b), confirming that both proteins activated the JAK‐STAT signalling pathway to very similar extents.

To obtain independent evidence for P‐hIL6 activity, we examined IL‐21 production in CD4^+^ T cells by observing activation of the JAK‐STAT signalling pathway following P‐hIL6 treatment (Dienz *et al*., 2009; Eddahri *et al*., 2009). CD4^+^ T cells were treated with either P‐hIL6 or E‐hIL6 at concentrations ranging from 3.125 to 25 ng/mL for 3 days. We measured IL‐21 levels and found that P‐hIL6 and E‐hIL6 had similar effects on IL‐21 production; both induced IL‐21 in a dose‐dependent manner (Figure 6c), indicating that plant‐produced hIL6 was biologically active.

## Discussion

We developed a highly efficient and cost‐effective method of purification of plant‐produced recombinant proteins. CBM3 and MCC beads were used as the affinity tag and affinity matrix, respectively, enabling the purification of proteins from plant whole‐cell extracts. The affinity tag was removed after purification to yield tag‐free hIL6. Using this approach, we were able to obtain approximately 18.5 μg hIL6 per g FW leaf tissue at near homogeneity, a moderate production level in compare to the heterologous protein production in the plant expression system (Chan and Daniell, 2015; Mortimer *et al*., 2015; Regnard *et al*., 2010; Schillberg *et al*., 2003).

Although a great deal of research has focused on the expression of genes in plants (Holtz *et al*., 2015; Tekoah *et al*., 2015), relatively little attention has been given to the downstream processing steps including cost‐effective purification and affinity tag removal. These downstream steps are, however, the major challenge facing industrial scale production of plant‐based recombinant proteins. Most of the purification protocols developed for protein expression in animal cells or bacteria can also be applied to proteins produced in plants. Plants, however, present unique problems, such as high levels of chloroplast proteins and cell wall components, and thus the development of protocols better suited to purifying proteins from plant cells is crucial if plants are to be used more widely as a platform for recombinant protein production. The easiest and most efficient method of protein purification involves use of an affinity tag. Most such tags are expensive, however, due to the high cost of the affinity matrix, limiting their use in industrial scale applications (Fong *et al*., 2010). We used CBM3, a CBD, as an affinity tag for protein purification. A large number of CBDs have been identified from various fungal and bacterial proteins (Linder and Teeri, 1996; Ong *et al*., 1989) and they have a high and specific affinity to cellulose, an inexpensive biomaterial (Wan *et al*., 2011; Wang and Hong, 2014; You and Zhang, 2013). Thus, an advantage of using the MCC as an affinity resin is the low cost. However, for other affinity resins such as protein A, Ni^2+^‐NTA, people have developed protocols to reuse them many times to reduce the cost of resins in protein purification. In this study, we did not test whether we can reuse the MCC beads to further reduce the cost of the affinity resin in protein purification.

An important consideration in selecting an affinity tag is that the tag must comprise a separate domain in the recombinant protein. Small affinity tags such as 6 × His, which consists of six histidine residues and is added to the C‐ or N‐termini, can be easily exposed at the protein surface to enable access to the affinity matrix for binding (Cheung *et al*., 2012). CBM3, with a molecular weight of 18 kDa, is much larger than the His tag. Like other CBDs, however, CBM3 forms a separate domain in its native protein (Wan *et al*., 2011; Wang and Hong, 2014) and it is therefore likely that, when fused to a target protein as an affinity tag, it will make up an independent domain in the fusion protein. We found that CBM3 placed in the middle of the MCS‐hIL6 fusion protein still exhibited tight binding to cellulose beads, suggesting that it formed an independent domain in the recombinant protein.

CBM3 binds to MCC beads. The cellulose fibre is composed of two alternating regions, the amorphous and crystalline regions (Bayer *et al*., 1998), and CBDs can be divided into two groups depending on whether they bind the amorphous or crystalline regions (Bayer *et al*., 1998; Ong *et al*., 1989). Earlier studies showed that most CBDs bind MCC irreversibly (Pinto *et al*., 2004; You and Zhang, 2013). We demonstrated the high binding affinity of CBM3 to MCC as it was not released by various treatments including 80% glycerol, ~40%–100% ethylene glycol and 0.1 m glycine. This tight binding allowed production of proteins with a high level of purity. The recombinant protein bound to MCC *via* CBM3 had a purity of over 95%, indicating that a single purification step yielded recombinant protein with sufficient purity for many applications.

It is in most cases highly desirable that target proteins do not contain any extra domains or residues. This can be achieved in two different ways; either only the target gene is expressed as a recombinant protein or the target protein is expressed as a fusion protein with other domains that are later removed during purification. We used the second approach, as purification of a protein without an affinity tag is a rather tedious procedure and can be highly expensive. Removal of extra domains relies on proteolytic cleavage and various proteases can be used to remove tags (Arnau *et al*., 2006; Waugh, 2011); TEV protease is one of the most popular proteases (Kapust *et al*., 2002). In addition, intein, a self‐cleavage sequence, has been used to remove extra domains fused to target proteins (Xu *et al*., 2000). Intein‐mediated self‐cleavage is induced after purification by specific conditions, including incubation in the presence of reducing agents such as dithiothreitol (DTT). Wan *et al*. (2011) used CBM3 to purify proteins expressed in *Pichia pastoris*. Their study examined whether intein‐based self‐cleavage could remove the affinity tag from CBM3‐containing proteins after immobilization on cellulose beads in a column. Intein‐based self‐cleavage, however, had low efficiency and required conditions of high temperature (16–37 °C), long incubation time (24–35 h) and a high concentration of a reducing agent (DTT or cysteine).

Most proteases have limited usefulness because they leave extra residues on the target proteins after cleavage. We therefore used a SUMO domain and SUMO‐specific protease to remove the extra domains from recombinant proteins; SUMO‐based proteolytic cleavage of the polypeptide is superior to other methods as it does not leave any extra sequence following cleavage at the di‐glycine residues of the recognition site (Frey and Görlich, 2014; Malakhov *et al*., 2004). Of the various proteases tested by Frey and Görlich (2014), bdSENP1 was the most efficient at removing the polyhistidine tag. We found that His:bdSENP1 recognized the bdSUMO domain in the middle of MCS‐hIL6 and efficiently cleaved the recognition site to release hIL6, either in solution or immobilized on MCC beads, from MCS‐hIL6. Most of the immobilized protein was cleaved by His:bdSENP1, indicating extremely efficient cleavage even when MCS‐hIL6 was bound to the beads. After cleavage, His:bdSENP1 was removed from the target proteins by Ni^2+^‐NTA affinity chromatography. The purity of target proteins can be improved by additional purification steps; we used size‐exclusion chromatography to obtain hIL6 with over 95% purity.

Endotoxin contamination is a big concern for recombinant proteins produced in bacteria such as *E. coli*. Endotoxin may induce severe toxic/inflammatory effects mediated by the TLR4 signalling pathway in mammals, including humans (Lu *et al*., 2008). In addition, minor traces of endotoxin can alter or hinder biological responses *in vitro* and *in vivo*; these changes include significant adverse effects on stem cell proliferation and differentiation (Lieder *et al*., 2013), and inhibition of cell growth and induction of programmed cell death (Munshi *et al*., 2002). Recently, Nomura *et al*. (2017) showed that endotoxins can adversely affect the proliferation ability of multipotent mesenchymal stem cells (MSCs). The lowest level of endotoxin with no adverse effect is 0.1 ng/mL, based on MSC proliferation (Nomura *et al*., 2017). An endotoxin‐free production platform of recombinant proteins is therefore highly desirable for cell‐based research and therapeutic applications. Plants contain no or very low levels of endotoxin (Magnusdottir *et al*., 2013); moreover, plants are free from animal cell‐infecting agents such as viruses and pathogens. Although we used *Agrobacterium*‐mediated transient expression to express hIL6 in *N. benthamiana*, the level of endotoxin in the purified protein was 0.2 EU/μg (0.02 ng/mL), which was much lower than the currently accepted safety limit [1 EU/μg (<0.1 ng/μg)] (Magnusdottir *et al*., 2013; Nomura *et al*., 2017). This confirmed that plant‐produced recombinant proteins contained low levels of endotoxin.

It is essential when producing recombinant proteins in heterologous systems that the target protein is biologically active. hIL6 activates the JAK‐STAT signalling pathway; hIL6 induces STAT3 phosphorylation in the human LNCaP cell line (Yu *et al*., 2014) and is able to promote IL‐21 production in activated CD4^+^ T cells (Dienz *et al*., 2009; Eddahri *et al*., 2009). We found that the activity of plant‐produced recombinant hIL6 was nearly identical to that of commercial hIL6 produced in *E. coli*, based on the level of STAT3 phosphorylation and induction of IL‐21 production, indicating that plant‐produced hIL6 was a biologically active protein.

In conclusion, we developed a CBM3‐based method to purify proteins from plant extracts and used SUMO domain and SUMO‐specific protease to remove the affinity tag. We also demonstrated its applicability by producing biologically active hIL6 in plants. This methodology has great potential for use in other plant biotechnology applications, especially for the production of plant‐based bioactive proteins.

## Experimental procedures

### Construction of plant and *E. coli* expression vectors

The *CBM3* (AEI55081), *bdSUMO* (amino acids 21–97) and *hIL6* (mature region, NM_000600) sequences were chemically synthesized (Bioneer corp., Daejeon, Korea). All synthetic genes were codon‐optimized for expression in *Nicotiana benthamiana*.

The chimeric construct *CBM3‐bdSUMO‐hIL6* was generated by overlapping PCR using primers PF‐1, PR‐2, PF‐3 and PR‐4 (Table S1). The ER retention signal HDEL (His‐Asp‐Glu‐Leu) was added to the C‐terminus of the coding sequence during overlapping PCR by incorporating the corresponding nucleotide sequence in the reverse primer. The final PCR products were digested with *BamHI* and *XhoI* restriction endonucleases and inserted into the plant expression vector 326‐GFP, digested with *BamHI* and *XhoI* (Kim *et al*., 2013). The M domain, the N‐terminal fragment from amino acid positions 231 to 290 of human protein tyrosine phosphatase receptor type C (CD45), was fused to the N‐terminus of *CBM3‐bdSUMO‐hIL6* by overlapping PCR using plasmid EelepfM (Kang *et al*., 2018) as a template and the primer sets PF‐5, PR‐6, PF‐7 and PR‐8 (Table S1). The plasmid obtained by PCR amplification, named *326*‐*M‐CBM3‐bdSUMO‐hIL6*, was digested with *PstI* and *EcoRI* and inserted into the plant expression binary vector pCAMBIA1300, digested with *PstI* and *EcoRI*, to give p1300‐C3bdSU‐hIL6 (Figure 1a). p1300‐C3bdSU‐hIL6 contained the double enhancer version of the cauliflower mosaic virus (CaMV) 35S promoter (d35S), 5′ untranslated enhancer region (5′ UTR) (Kim *et al*., 2013), the BiP leader sequence (amino acid positions 1 to 34) from *Arabidopsis thaliana* BiP1 (BAA13947) and the HSP terminator from *Arabidopsis thaliana* (Nagaya *et al*., 2010).

To express His:bdSENP1 in *Escherichia coli*, a chemically synthesized (Bioneer Corp., Daejeon, Korea) catalytic region from *bdSENP1* (amino acid residues 242–481) was digested with *BamHI* and *EcoRI* restriction endonucleases and inserted into pRSET‐A (Invitrogen, Carlsbad, CA), digested with *BamHI* and *EcoRI*, to give pRSET‐bdSENP1 (Figure S2A). The nucleotide sequences of all constructs were confirmed by DNA sequencing (Macrogen, Korea).

### Expression and purification of bdSENP1 in *E. coli*

*E. coli* BL21 (DE3) pLysS cells (New England Biolabs Inc., Hitchin, England) were transformed with the construct *pRSET‐bdSENP1*. An overnight pre‐culture was prepared with a single colony from the transformation, and 1 mL culture was used to inoculate 400 mL LB medium (CONDA, Italy, cat. no. 1551.00) containing 50 mg/mL ampicillin sodium (Affymetrix/USB™, cat. no: 69‐52‐3) in a 1 L flask. The culture flask was incubated at 37 °C with shaking (225 rpm) until OD_600_ reached 0.4–0.6. IPTG (Duchefa Biochemie, Haarlem, Netherlands) was immediately added to a final concentration of 1 mm to induce expression, and cells were incubated at 37 °C for 4 h. The cells were centrifuged at 12 000 ***g*** at 4 °C for 10 min. The harvested cells were resuspended in 30 mL cold lysis buffer (40 mm Tris‐HCl, pH 7.5, 300 mm NaCl, 1 mm DTT, 20 mm Na_2_PO_4_, 1% Triton‐X‐100, 10 mm imidazole and protease inhibitor cocktail (Roche, Mannheim, Germany) and lysed by sonication (Fisher Scientific Sonic Dismembrator Model‐500) on ice for 2 s, followed by 3 s pulses for 10 min. Finally, lysates were clarified by centrifugation at 15 000 ***g*** at 4 °C for 30 min and proteins were purified from the supernatant using a Ni^2+^‐NTA agarose column (Qiagen, Valenica, CA), according to the manufacturer's instructions.

### Transient expression in *N. benthamiana*

The binary plant expression vector p1300‐C3bdSU‐hIL6 was transformed into *Agrobacterium tumefaciens* strain GV3101. Separate cultures of *Agrobacterium* harbouring p1300‐C3bdSU‐hIL6 and *Agrobacterium* harbouring p38 were grown overnight in YEB liquid medium. *Agrobacterium* cells were collected by centrifugation at 3500 ***g*** for 8 min and resuspended in infiltration buffer (10 mm MES, 10 mm MgSO_4_, 100 μm acetosyringone, pH 5.6) to reach OD_600_ of 0.8 and mixed each in a 1 : 1 ratio (v/v). Leaf tissues of 5 to 7‐week‐old *N. benthamiana* plants were co‐infiltrated with the *Agrobacterium*‐mixture of suspension cells (Marusic *et al*., 2016). Plants were returned to the greenhouse and grown for a further 3 to 7 DPI.

Fresh leaf tissues were ground under liquid nitrogen to a fine powder in protein extraction buffer (50 mm Tris‐HCl, pH 7.5, 150 mm NaCl, 1 mm DTT, 0.1% [v/v] Triton X‐100 and protease inhibitor cocktail). Total soluble proteins (TSP) were extracted from the ground tissue samples. Lysates were clarified by centrifugation (13 000 ***g***) for 30 min and total protein concentrations were measured using the Bradford protein assay (Bio‐Rad, Hercules, CA).

### SDS‐PAGE and Western blotting

Proteins were separated using 10%–12.5% sodium dodecyl sulphate‐polyacrylamide gel electrophoresis (SDS‐PAGE). Proteins were transferred to membranes and either stained with 0.25% CBB R‐250 (AMRESCO, cat. no: 6104‐59‐2) in a staining solution containing 45% methanol and 10% glacial acetic acid or analysed by Western blotting with appropriate antibodies.

For Western blot analysis, membranes were blocked with 5% non‐fat‐dried milk in TBST buffer (20 mm Tris‐HCl, pH 7.5, 500 mm NaCl, 0.05% Tween‐20) for 2–3 h, washed three times with TBST, and incubated with mouse anti‐penta‐His (Qiagen, Valenica, CA), mouse anti‐IL6 (abcam, ab9324), rabbit anti‐CBM3 (Bioapp., Korea), anti‐STAT3 (Santacruz, sc‐482), anti‐p‐STAT3 (Santacruz, sc‐8052) or anti‐β‐actin (Santacruz, sc‐47778) antibodies at a dilution of 1 : 1000 in TBST with 2.5% non‐fat dry milk for 2 h. Immunoblotting bands were visualized using enhanced chemiluminescence (ECL kit; Amersham Pharmacia Biotech, Buckinghamshire, UK) and images were obtained with a LAS 4000 image capture system (FUJIFILM, NJ).

### CBM3‐based affinity purification and removal of the affinity tag

To purify MCS‐hIL6 using MCC beads, protein extracts were mixed with MCC powder (Sigma‐Aldrich, St. Louis, MO, CAS Number 9004‐34‐6) in a batch mode. Briefly, 700 mg MCC beads were suspended in four‐bead volumes of water and the supernatant containing the fine cellulose particles was removed. This procedure was repeated five times until the supernatant was clear. The MCC beads were resuspended with water at 1 : 1 ratio (v/v) (~2.1 mL bead‐volume) and 80 mL TSP (40 g FW) was divided between two 50 mL centrifuge tubes (40 mL in each tube). Each tube was mixed with half of the MCC slurry and placed at 4 °C with gentle shaking (60 rpm) to allow binding. After incubation for 1 h, the samples were centrifuged at 2000 ***g*** for 2 min, and the supernatant (unbound fraction) and MCC beads with bound proteins were collected separately. The MCC beads were washed four times with four‐bead volumes of 40 mm Tris‐HCl buffer (pH 7.5) to remove loosely bound protein. To determine purity, bound protein was released from the beads by boiling in 2× SDS‐PAGE buffer (100 mm Tris‐HCl, pH 6.8, 4% (w/v) SDS, 0.1% (w/v) bromophenol blue, 20% (v/v) glycerol, 200 mm β‐mercaptoethanol) and analysed *via* SDS‐PAGE followed by CBB staining.

To remove the affinity tag from recombinant MCS‐hIL6, MCC beads with bound MCS‐hIL6 were washed once in His:bdSENP1 reaction buffer (25 mm Tris‐HCl buffer, pH 7.5, 0.1% NP‐40, 250 mm NaCl and 1 mm DTT) and incubated with His:bdSENP1 protease (10 μg) in 10 mL reaction buffer for 6 h at 4 °C with gentle shaking (60 rpm). The supernatant containing the released hIL6 was collected. His:bdSENP1 in the supernatant, together with hIL6, was removed using a Ni^2+^‐NTA agarose column. Finally, purified hIL6 was concentrated using a centriprep 10K centrifugal filter (Millipore Korea, cat. no. 4304).

### Size‐exclusion chromatography

hIL6, previously purified using MCC beads, was further purified on a HiLoad 16/600 Superdex 200 pg column using a fast protein liquid chromatography (FPLC) system (AKTA purifier GE Healthcare systems) at 4 °C in 1× PBS. Proteins were eluted in PBS at a flow rate of 0.4 mL/min (Villani *et al*., 2009). Protein concentration was measured at 280 nm. Peaks representing hIL6 fractions were collected and concentrated using a centriprep 10K centrifugal filter (Millipore Korea, cat. no. 4304).

### Enzymatic deglycosylation of purified recombinant hIL6

Purified hIL6 was subjected to deglycosylation using endo‐H (Roche, USA). Briefly, 4 μg purified hIL6 was mixed with 1× glycoprotein denaturing buffer (0.5% SDS and 1 mm dithiothreitol). The mixture was boiled at 100 °C for 10 min, cooled to room temperature and then treated with 0.5 units of endo‐H at 37 °C for 2 h.

### Determination of endotoxin levels

Endotoxin levels in purified hIL6 were determined using the Pierce™ LAL Chromogenic Endotoxin Quantitation Kit (Thermo Fisher Scientific, Rockford, Cat. no. 88282).

### Biological activity of hIL6

The biological activity of plant‐produced hIL6 was determined using phosphorylation of STAT3. LNCaP cells were seeded at 0.8 × 10^6^ cells in a 60 mm culture dish and incubated for 1 day. The culture medium was substituted with Roswell Park Memorial Institute (RPMI) medium 1640, which did not contain 10% FBS, and incubated for 8 h. Cells were treated with plant‐produced hIL6 or *E. coli*‐produced hIL6 (Perprotech, 200‐06). Cells were harvested 2, 5, 15, 30 and 60 min after hIL6 treatment and washed with PBS. Cells were lysed in radioimmunoprecipitation assay (RIPA) buffer (Thermo Fisher Scientific), which contained 1× protease inhibitor (Roche, complete ULTRA tablets), and proteins were extracted. Protein concentration was quantified using a BCA assay kit (Thermo Pierce). Equal amounts of protein samples were separated using 8% SDS‐PAGE and analysed by immunoblotting.

To measure IL‐21 production in CD4^+^ T cells after IL6 treatment, splenocytes from C57BL/6 mice (Jackson Laboratory) were isolated and live CD4^+^ T cells were sorted using a FACS sorter (Moflo, Beckman Coulter). Sorted cells (10^5^ per well) were cultured in flat‐bottom 96‐well plates (Costar, Corning) coated with 5 μg/mL of anti‐CD3 antibody and 2.5 μg/mL of anti‐CD28 antibody. RPMI medium supplemented with 10% FBS (Hyclone), 1× penicillin and streptomycin and 50 μm beta‐mercaptoethanol was used for cell culture. hIL6 produced in plants (this study) or in *E. coli* (R&D Biosystems) was added to each well. IL‐21 levels in the supernatants were measured after 3 days of incubation by ELISA, according to the manufacturer's instructions (mouse IL‐21 duoset ELISA, R&D Biosystems).

## Conflict of interest

The authors have no financial conflicts of interest to report.

## Supporting information


**Figure S1** Binding capacity of CBM3 fusion protein on microcrystalline cellulose (MCC).
**Figure S2** Expression and purification of His:bdSENP1 in *Escherichia coli*.
**Figure S3** Size‐exclusion column chromatography.
**Figure S4** Quantification of purified hIL6.
**Figure S5** hIL6 purified from plant extracts contains a low level of endotoxin.
**Table S1** Primers used in this study.
