# Supplementary material for: Cost‐effective production of tag‐less recombinant protein in Nicotiana benthamiana
Source: Plant Biotechnol J. 2018 Dec 8;17(6):1094–105. doi: 10.1111/pbi.13040 (PMC6523591; doi:10.1111/pbi.13040)
Supplement: Supplementary file 1 — Figure S1 Binding capacity of CBM3 fusion protein on microcrystalline cellulose (MCC). Figure S2 Expression and purification of His:bdSENP1 in Escherichia coli. Figure S3 Size‐exclusion column chromatography. Figure S4 Quantification of purified hIL6. Figure S5 hIL6 purified from plant extracts contains a low level of endotoxin. Table S1 Primers used in this study. [file PBI-17-1094-s001.docx]

**Cost-effective production of tag-less recombinant protein in *Nicotiana benthamiana***

Md Reyazul Islam^1^, Ju-Won Kwak^1^, Jeon-soo Lee^2^, Sung-Wook Hong^2^, Md Rezaul Islam Khan^1^, Yongjik Lee^1^, Yoontae Lee^2^, Seung-Woo Lee^2^, and Inhwan Hwang^1^**^*^**

^1^Division of Integrative Biosciences and Biotechnology, Pohang University of Science and Technology, Pohang, 37673, South Korea.

^2^Department of Life Science, Pohang University of Science and Technology, Pohang, 37673, South Korea.

***Correspondence:** Inhwan Hwang

Division of Integrative Biosciences and Biotechnology, Pohang University of Science and Technology, Pohang, 37673, South Korea.

Tel: +82-54-279-2128

E-mail: [ihhwang@postech.ac.kr](mailto:ihhwang@postech.ac.kr)

**Supplementary Figures**


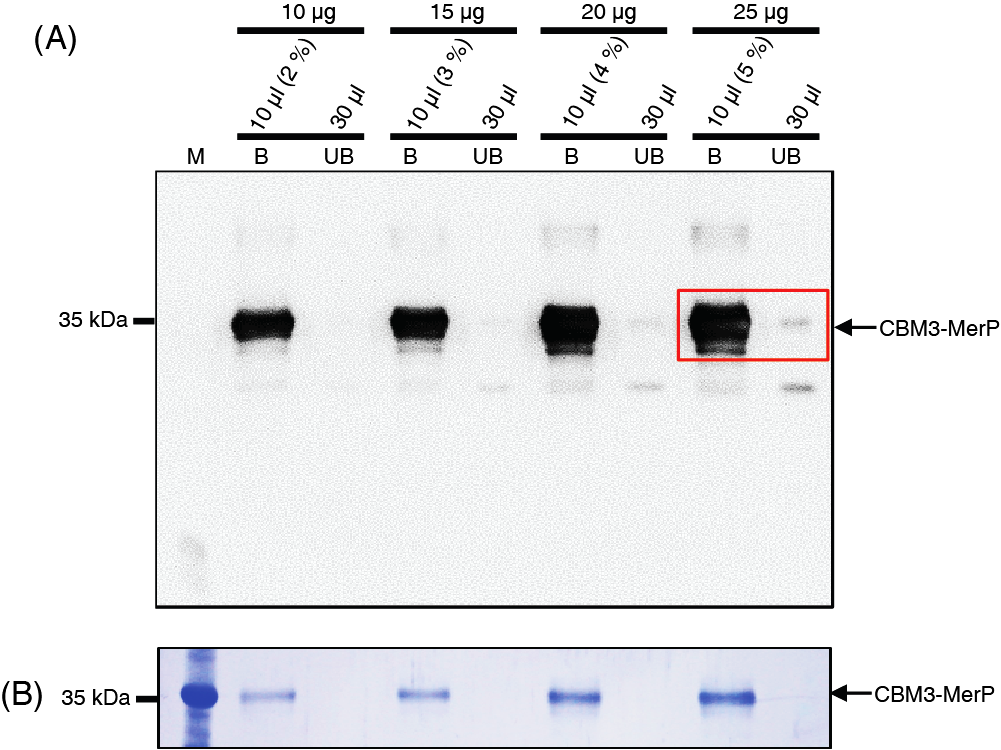


**Supplementary Figure S1. Binding capacity of CBM3 fusion protein on microcrystalline cellulose (MCC).**

To determine the binding capacity of CBM3 on MCC beads, different amounts (10–25 µg) of CBM3-MerP produced in *E. coli* were incubated with 10 mg MCC beads. After binding, the MCC beads and the supernatant (unbound fraction) were collected separately. The MCC beads were washed four times with 40 mM Tris-HCL (pH 7.5). Proteins bound to the beads (bound fraction) were released by boiling in 500 μl of 2 × SDS-reducing buffer (100 mM Tris-HCl, pH 6.8, 4% (w/v) SDS, 0.1% (w/v) bromophenol blue, 20% (v/v) glycerol, and 200 mM β-mercaptoethanol). Proteins in 10 µl (2–5% of the total volume) of the bound fraction (B) or 30 µl (6% of the total volume) of the unbound (UB) fraction were separated by 12% SDS-PAGE and analyzed by western blotting with anti-CBM3 antibody (**A**) or CBB staining (**B**). It was found that 25 µg CBM3:MerP slightly oversaturated 10 mg MCC beads as this concentration resulted in a small quantity of protein in the unbound fraction. It was therefore concluded that 10 mg MCC beads (0.03 mL bead-volume) had a binding capacity of 20 µg CBM3-MerP, corresponding to 2 mg CBM3-MerP per gram MCC beads or 2 mg CBM3-MerP per 3 mL bead-volume.


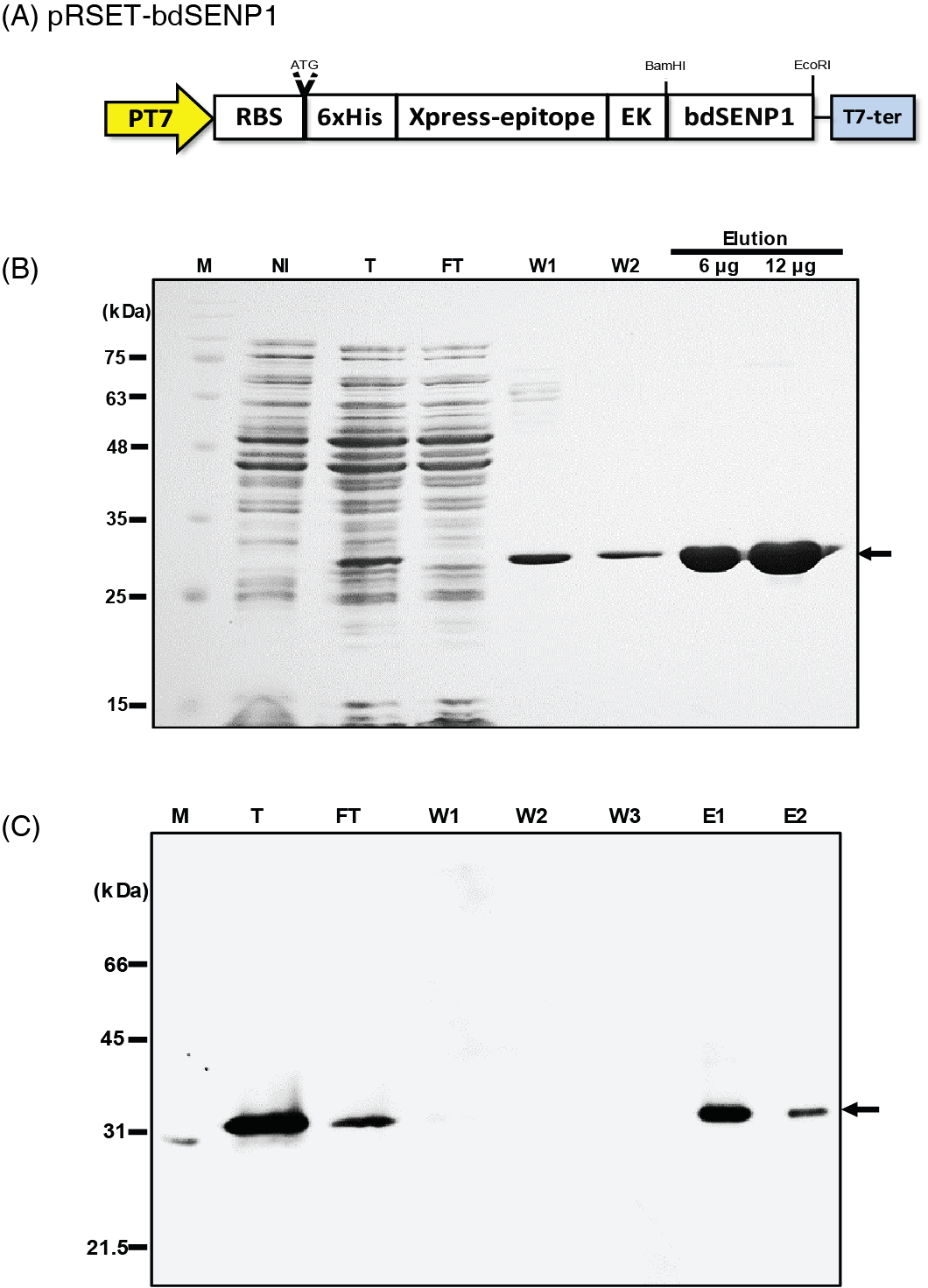


**Supplementary Figure S2. Expression and purification of His:bdSENP1 in *Escherichia coli*.**

*E. coli* BL21 (DE3) cells were transformed with the expression construct *pRSET-bdSENP1* (**A**) and IPTG was used to induce expression of His:bdSENP1. His:bdSENP1 was purified from total protein extracts using Ni^2+^-NTA affinity column chromatography. Proteins in the samples were separated by SDS-PAGE and analyzed by staining with CBB (**B**) or by western blotting with anti-His antibody (**C**). M: pre-stained molecular weight standards; T: total crude extracts from IPTG-induced *E. coli*; NI: total crude extracts from non-induced *E. coli*; FT: flow-through fraction; W1 to W3: wash-off fractions; E: elution fraction. Arrows indicate the position of His:bdSENP1 (31 kDa). Quantitative analysis of purified His:bdSENP1 was performed using the Bradford protein assay with bovine serum albumin (BSA) as a standard.


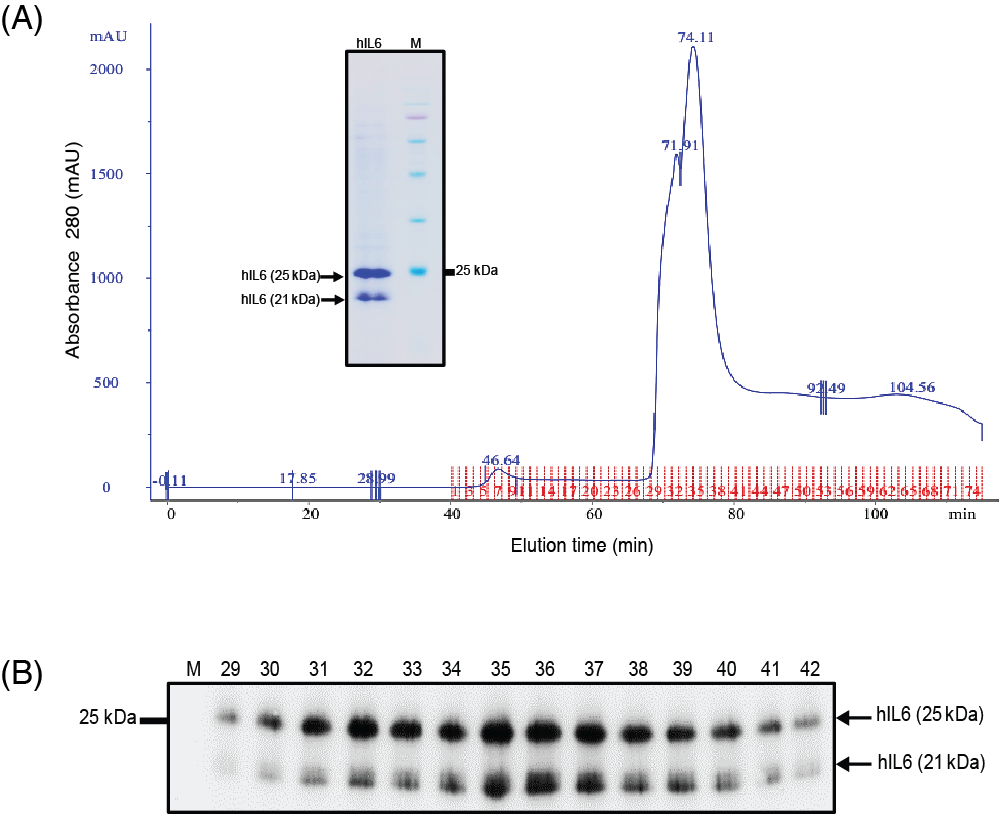


**Supplementary Figure S3. Size-exclusion column chromatography.**

The hIL6 fraction obtained by Ni^2+^-NTA affinity column chromatography was loaded onto a size-exclusion gel filtration column and 1 mL fractions were obtained by FPLC chromatography. The elution profile was obtained at 280 nm (**A**). The fractions were analyzed by western blotting using anti-hIL6 antibody (**B**). The protein fractions used for gel filtration column chromatography were analyzed by SDS-PAGE and stained with CBB (inset in **A**).


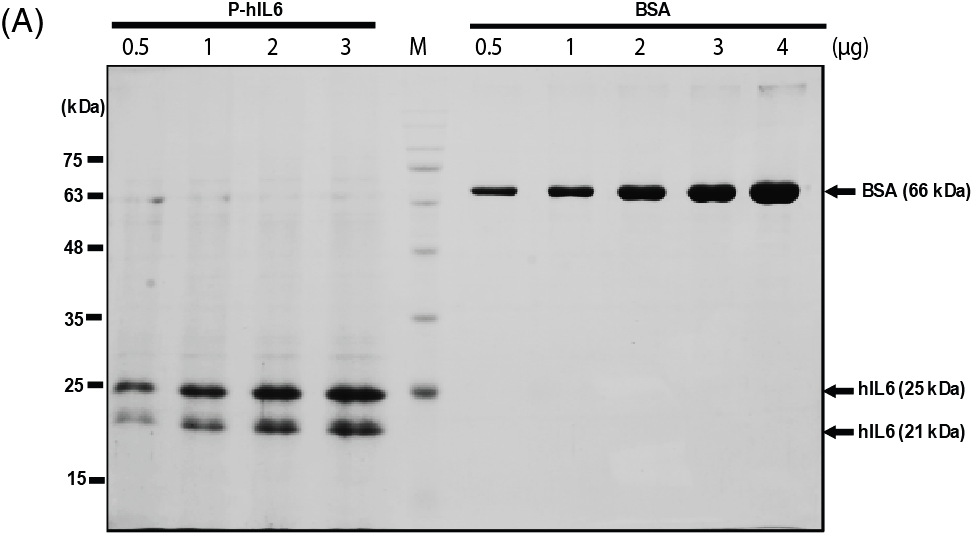


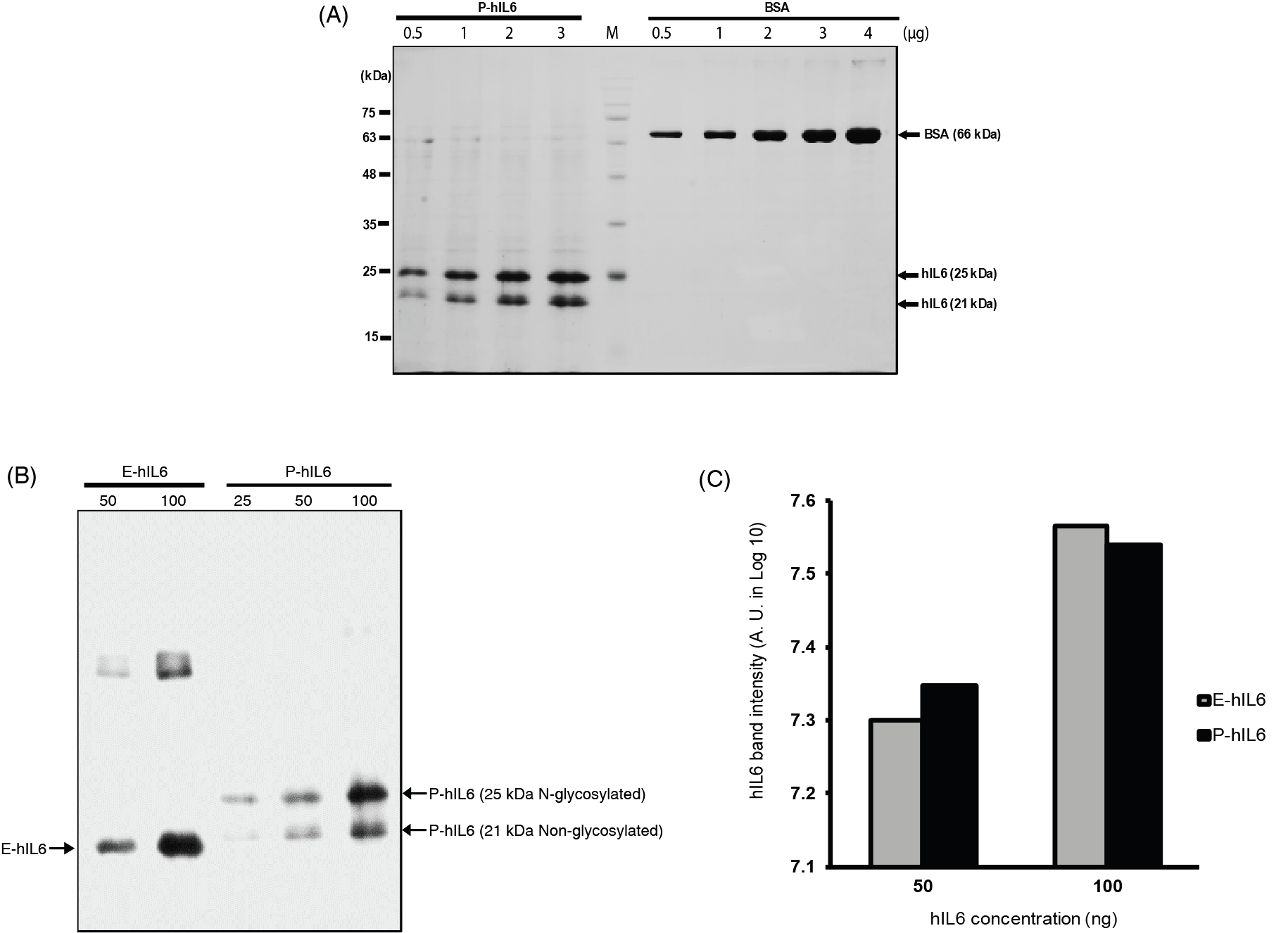


**Supplementary Figure S4. Quantification of purified hIL6.**

**(A)** Quantification of P-hIL6. The amount of purified hIL6 (P-hIL6) was first measured using the Bradford protein assay. To confirm this quantification, different amounts (as indicated on the figure) of P-hIL6 and bovine serum albumin (BSA) were separated by 12.5% SDS-PAGE followed by CBB staining (**A**). The signal intensity was used to compare quantities of P-hIL6 and BSA. M: protein standards.

(**B, C**) Quantification of P-hIL6 and commercial *E. coli*-produced hIL6 (E-hIL6). The indicated quantities of proteins were separated by SDS-PAGE and analyzed by western blotting with anti-IL6 antibody (**B**). The signal intensity of the P-hIL6 and E-hIL6 bands was measured using Multi Gauge V2.2 densitometric software (Fujifilm), which determines the quantity of hIL6 in arbitrary units (A.U.) on a base 10 log scale (Log_10_). Quantities of N-glycosylated and non-glycosylated P-hIL6 were combined to give the final concentration; the concentration of E-hIL6 was that provided by the manufacturer (**C**).


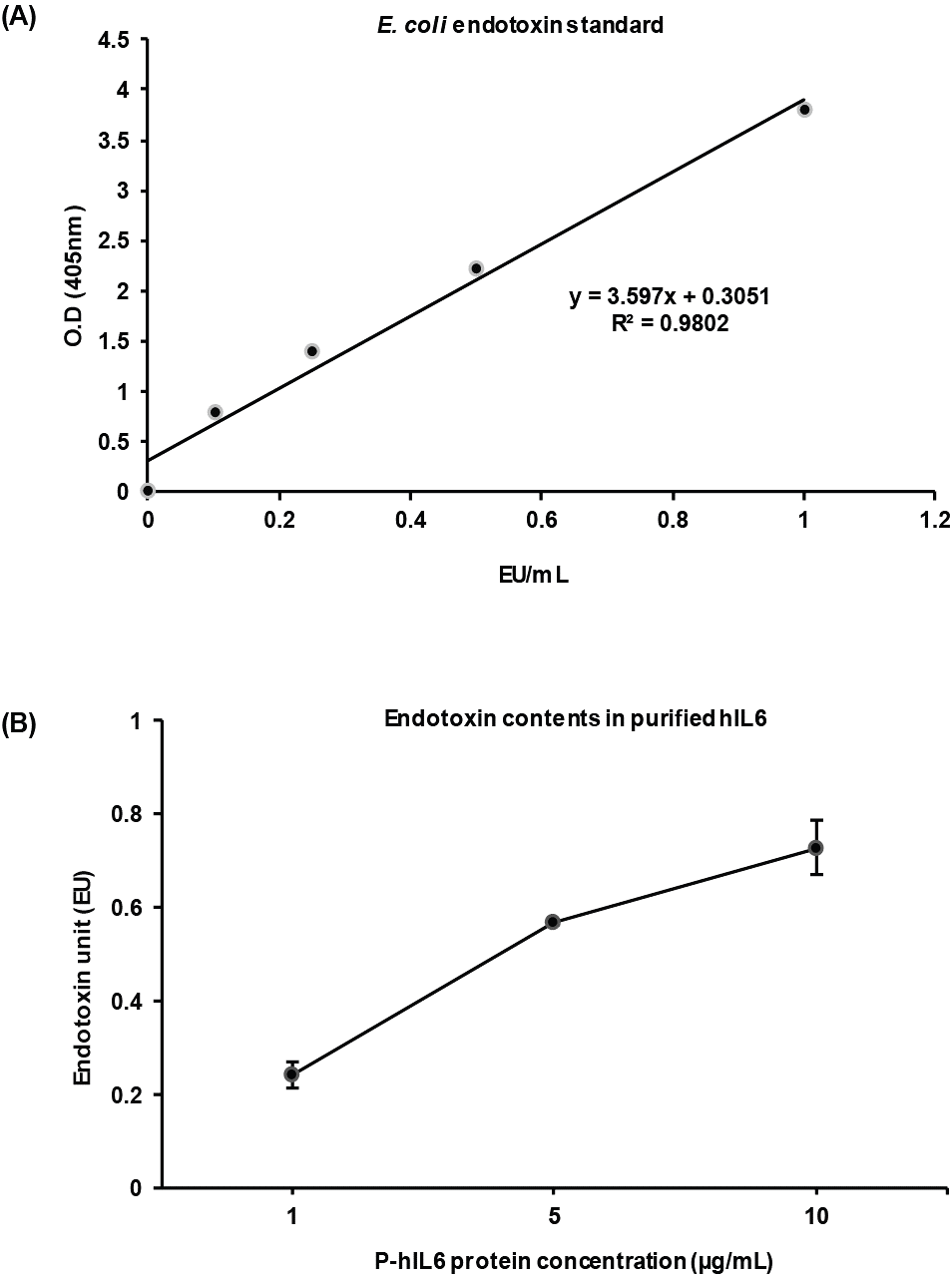


**Supplementary Figure S5. hIL6 purified from plant extracts contains a low level of endotoxin.**

The amount of endotoxin in purified hIL6 was measured using the LAL Chromogenic Endotoxin Quantitation Kit (Pierce). The standard curve was generated using a commercially available *E. coli* endotoxin standard (**A**). Varying amounts (1 to 10 µg) of plant-produced hIL6 (P-hIL6) were used to determine the endotoxin content of P-hIL6 (**B**) by chromogenic endotoxin quantitation. Data are means (n = 3); error bars show the standard deviation.

**Supplementary Table S1:** **Primers used in this study.**

| **Primer** | **Sequence 5’-3’** |
| --- | --- |
| PF-1 | CGGGATCCCGGTATCAGGTAACCTTAAGG |
| PR-2 | CCTCTCCTGGAGGAACCATACCACCAGTCTGATGTAAC |
| PF-3 | GTTACATCAGACTGGTGGTATGGTTCCTCCAGGAGAGG |
| PR-4 | CCGCTCGAGCTAGAGCTCATCGTGCATCTGCCTAAGAGCCCT |
| PF-5 | CGGGATCCCGATGGCAAACATCACTGTGGATTAC |
| PR-6 | CTTAAGGTTACCTGATACTGATCCACCACCAGACCC |
| PF-7 | GGGTCTGGTGGTGGATCAGTATCAGGTAACCTTAAG |
| PR-8 | CCCCCGGGGACCAGGTTCCTTTCCCCACAC |
